# Communal coping among African Americans: a comparison of romantic and non-romantic dyads

**DOI:** 10.1007/s10865-026-00658-2

**Published:** 2026-03-27

**Authors:** Vicki S. Helgeson, Abigail Vaughn, MarQia Allen

**Affiliations:** 1 University of Pittsburgh, Pittsburgh, PA 15213 USA; 2 North Carolina State University, Raleigh, USA

**Keywords:** Communal coping, Shared appraisal, Collaboration, Type 2 diabetes, Black persons with diabetes, Health

## Abstract

There is a great deal of research linking communal coping to good relationship and health outcomes. However, this research has neglected to examine dyads other than romantic couples, and research on underrepresented groups is sparse. The goal of this exploratory study was to compare communal coping (shared appraisal, collaboration) among Black persons with romantic partners to Black persons with other close relationship partners. First, we compared levels of communal coping and outcomes between the two groups. Romantic dyads reported greater communal coping and greater support provision than non-romantic dyads, but non-romantic dyads reported greater intimacy than romantic dyads. Second, we examined the implications of the two components of communal coping for relationship and health outcomes and tested whether these relations were the same for the two groups. Results showed links of own communal coping to better relationship outcomes for both patients and dyad partners. There was some suggestion that these beneficial links are stronger among romantic than non-romantic couples, and that patients may benefit in particular from dyad partner communal coping. There was some evidence for benefits of communal coping on psychological health, but little evidence for benefits on diabetes outcomes—and these findings generalized across both groups.

Type 2 diabetes is one of the most pervasive and costly chronic illnesses in the United States in terms of both psychological and physical health (Centers for Disease Control and Prevention, 2024). Much of the research in this area has focused on characteristics of the person with type 2 diabetes (T2D) and how they manage the disease in terms of taking medication, making dietary changes, and implementing a physical activity plan. However, disease management takes place in an interpersonal context that extends beyond the patient: the disease affects social network members, and social network members have opportunities to help or hinder T2D management. There is a large literature that shows social support is related to the self-management of chronic illness—especially chronic illnesses that are associated with complex regimens such as diabetes (Gallant et al., 2007; Ruddock et al., 2016). With the escalating rate of type 2 diabetes and health care resource limitations, practitioners must recognize the importance of the family in helping those with diabetes take care of themselves. Previous qualitative (e.g., Beverly & Wray, 2010), correlational (e.g.,Trief et al., 2004) and intervention studies with persons with diabetes (see Van Dam et al., 2005 for a review) have suggested that spouse support may enhance adherence and alter health behaviors. However, these studies often lack a theoretical framework as to how social network members help or hinder self-care.

Over the past 10 years, we adopted a communal coping perspective and applied it to the understanding of the partner role in patient adjustment to type 2 diabetes (see BLINDED for a review). Communal coping is defined as appraising a stressor as shared with a partner and collaborating with that person to manage the stressor (Helgeson et al., 2018; Lyons et al., 1998). Although there is a wealth of evidence that communal coping with a partner is beneficial for health in the context of cancer (Berg et al., 2008), diabetes (Zajdel & Helgeson, 2020), and other health problems (Rentscher, 2019), much of this research has been conducted in the context of romantic relationships. An exception is research that has examined communal coping in the context of stressors that affect communities, such as natural disasters (W. A. Afifi et al., 2012) (W. A. Afifi et al., 2012) and people living in refugee camps (T. D. Afifi et al., 2016), which also has shown links to good adjustment. Nonetheless, we do not know if communal coping with persons other than romantic partners is equally beneficial.

Another limitation of previous research on communal coping is that the majority of studies has focused on White persons. One study showed that the relation between communal coping and self-care was stronger among Black than White individuals with type 2 diabetes (Basinger & Hartsell, 2020). Another study compared the links of communal coping to health among both Black and White patients and spouses, finding that White patients and Black spouses benefited more than Black patients and White spouses (Helgeson et al. 2019a, b). As findings are not definitive and research is sparse in this area, more research needs to be conducted on underrepresented groups.

There is reason to believe that communal coping might operate differently among African Americans compared to White persons, as research has shown that African Americans rely more on network members other than spouses in coping with diabetes compared to Whites. African Americans receive a great deal of instrumental support (e.g., transportation, childcare) from family members—especially in the context of illness, and even when they are married (Cross et al., 2018). There is some evidence that African Americans with lower levels of education and income are the most likely to turn to family for support (Cross et al., 2018). Extended family are not only central to the lives of low income African Americans but also low income persons from other racial/ethnic groups including White persons, making social class more important than race (Gerstel, 2011). This situation is described nicely by Dixon (2009) “The reason that marriage might not be as central to African Americans as it is for White Americans is that they have extended family networks to insulate them in the face of economic and demographic hardships that impede their ability to form stable marriages and families” (p. 35). One study compared support from friends and family across African Americans, Black Caribbeans, and NonHispanic Whites and found that Black people were more likely to interact with and exchange support with family compared to the other groups, whereas White people were more likely to interact with and exchange support with friends (Taylor et al., 2013). In a follow-up study, both African Americans and Black Caribbeans provided and received more support from extended family compared to NonHispanic Whites (Taylor et al., 2022). This finding generalized across income groups, suggesting the differences were due to race rather than social class. Family itself is also defined somewhat differently in the African American community extending beyond those who are related by blood to other close relations, known as fictive kin (Dilworth-Anderson, 1992). Unfortunately, most research on family relationships focuses on the spouse and fails to consider relationships with extended family or fictive kin. We hope to rectify this limitation here.

To address some of the limitations in the work on communal coping, we conducted an exploratory study of a sample of Black persons with T2D and their romantic partners (follow-up from a previous study) and compared them to a newly recruited sample of Black persons with T2D who identified a significant support person who was not a romantic partner. Our primary goal was to compare communal coping among Black persons with romantic partners to communal coping among Black persons with other support sources. First, we examined if there were group differences in communal coping. Second, we examined if the link of communal coping to relationship and health outcomes was the same for patients and partners in both groups. Because our sample sizes for the two groups were small (*n*’s 41 and 34), we considered the study to be exploratory and a first step in examining these questions.

## Method

### Participants

We followed a previously enrolled community sample of persons with T2D (*n* = 207) for five years. Eligibility requirements for the original sample were type 2 diabetes diagnosis within the past 5 years, no other illness that affected daily life more than diabetes (e.g., cancer), and married/cohabiting with a romantic partner who did not have diabetes and was willing to participate in the study. The sample was recruited using mass media advertising, mass transit advertising, brochures in physician offices, postings in community centers and churches, and the University of Pittsburgh Medical Center research registry. Interested persons contacted the team and were screened for eligibility. The majority were determined not to be eligible (419 out of 658), largely because they were diagnosed more than 5 years ago. Of the 239, 4 refused before we could determine eligibility, 22 refused after being screened as eligible, 3 were found to be ineligible after signing the consent form, and 3 couples were dropped from the analyses (1 was intoxicated during the study, 1 couple was determined not to be romantic partners, 1 participant had type 1 diabetes instead of type 2 diabetes), leaving a final initial sample of 207. Of these 207 couples, 55% were male and 45% were female; 53% were White and 47% were Black; and median income ranged between $40–59,000. Participants in this original sample had been diagnosed with T2D on average 1.88 (*SD* = 1.68) years before the study began. The subject of the present article is on the five year follow-up data from the Black subsample. At follow-up, we retained 58 (60%) of the Black persons with T2D. However, we could only include the 41 persons with T2D who had a romantic partner who was willing and able to participate in the study (10 separated/divorced, 2 partner died, 3 partner refused, 2 partner’s data was not usable due to cognitive issues).

At follow-up, we recruited a new comparison sample of 34 unmarried Black persons with T2D who identified a friend or family member who was willing to participate in the study. Eligibility requirements were over age 18, African American race, diagnosed with T2D between 5 and 15 years ago (to match the original other sample), no major chronic illness that affects life more than diabetes, and unmarried and not living with a romantic partner. The person they nominated to be their dyad partner in the study could not have diabetes and must be willing to participate in the study. Dyad partners were mostly friends, children, and other family members. Demographics for both groups are shown in Table 1.

### Procedure

We obtained Carnegie Mellon Institutional Review Board (8/11/2017) approval for all study procedures. The romantic couple sample was recruited from the original study. The non-romantic sample was recruited with the same procedures as described above for the romantic sample.

We obtained informed consent from both members of the dyad prior to the start of any study procedures. The follow-up session was conducted in-person until the COVID-19 pandemic emerged. After stay-at-home orders were issued on March 16th, 2020, we conducted the remaining interviews remotely via zoom. Although we had been interviewing both groups at the same time, when the pandemic occurred we had recruited and interviewed the entire nonromantic sample but completed the remaining 37% of the interviews with the romantic sample via zoom. Couple members completed surveys and were interviewed independently, followed by a videotaped discussion about diabetes. Participants were paid $50 for this session.

### Instruments

#### Background variables

We asked each person to report general demographic information: gender, race, ethnicity, education, income; and we asked persons with T2D to report their date of diagnosis with T2D and their medication regimen (e.g., oral medication, insulin). Participants completed a brief comorbidity questionnaire, which was developed by Katz et al. (1996) and based on the Charlson Index (1987). To assess family income, we provided a response scale in 10 K increments starting with 20,000 or less and ending with over 100,000.

#### Communal coping

The appraisal aspect of communal coping was measured with a single item that has been used widely in previous research (Helgeson et al., 2018): “When you think about problems related to your diabetes, to what extent do you view those as ‘our problem’ (shared by you and your partner equally) or mainly your own problem?” Response scale ranged from 1 (completely my problem) to 5 (both of our problem). The collaboration aspect of communal coping consisted of four components. First, we asked “When a problem related to diabetes arises, how much do you and your partner work together to solve it?” (response scale 1 = none of the time; 5 = all of the time). Second, we provided the adapted Inclusion of Other in Self for Diabetes Scale (Helgeson & Van Vleet, 2019), which is a pictorial representation of the self and partner in connection with diabetes. Respondents are asked to choose from a set of 7 circles that vary in their degree of overlap as to which best represents how they and their partner deal with diabetes. Third, we asked how involved the partner was in diabetes on a 5-point scale ranging from 1 = not at all to 5 = a lot. Fourth, we asked 3 items about collaboration (Berg et al., 2020) and took the average (α = 0.87 and 0.86 for patient and partner respectively): You and your partner work together to manage diabetes; You and your partner discuss how to best manage diabetes; You and your partner make decisions together about how to handle diabetes. The internal consistency of these four components was 0.81 for patients and 0.86 for partners. Thus, we standardized the items and took the average to form a collaboration index. The correlation between appraisal and collaboration was moderate for patients (*r* = 0.46, *p* < 0.001) and slightly higher for partners (*r* = 0.60, *p* < 0.001).

#### Relationship outcomes

We adapted the 5-item Quality of Marriage Index (QMI; Norton, 1983; α = 0.94 and 0.96 for patient and partner respectively) for couples who were not married or involved in a romantic relationship (i.e. “We have a good marriage” changed to “We have a good relationship”) and administered the 6-item emotional intimacy subscale from the Personal Assessment of Intimate Relationships (PAIR) scale (Schaefer & Olson, 1981; α = 0.81 and 0.87 for patient and partner respectively). We used the 5-item emotional support and 3-item instrumental support scales from previous research (patient α = 0.76 and 0.84; partner α = 0.79 and 0.83; Helgeson et al., 2017). Participants responded to all of the support items on a 4-point scale: 0 = none, 1 = a little, 2 = some, and 3 = most. Because the emotional and instrumental support scales were so highly correlated in this study (patient *r* = 0.69, *p* < 0.001; partner *r* = 0.64, *p* < 0.001), we averaged the two to create a support index.

#### Psychological outcomes

We administered three measures of psychological distress to patients and partners. First, we administered the Center for Epidemiological Depression Scale (Radloff, 1977) to measure depressive symptoms (α = 0.92 and 0.87 for patient and partner respectively). Second, we used Diener’s Life Satisfaction Scale ((Diener et al., 1985; α = 0.87 and 0.86 for patient and partner respectively). Third, we administered the 4-item abbreviated Perceived Stress Scale (Cohen et al., 1983; α = 0.73 and 0.76 for patient and partner respectively). Because these three variables were strongly related (*r*’s ranged from − 0.53 to 0.78, all *p*’s < 0.001), we reverse scored depression and perceived stress, standardized the three variables, and took the average to form a psychological health index.

#### Patient DM outcomes

We measured diabetes distress with the 17-item Diabetes Distress Scale (Polonsky et al., 2005), which taps emotional burden, physician-related distress, regimen-related distress, and interpersonal distress (α = 0.91). We measured self-care behavior with the Summary of Diabetes Self-Care Activities (Toobert & Glasgow, 1994), which measures dietary intake, exercise/energy expenditure, and medication adherence. The reliability was good (α = 0.71). We also used the 4-item Medication Adherence Scale (Morisky et al., 1986) which had good reliability (α = 0.81). We used the DCA Vantage analyzer to measure HbA1c for interviews conducted in person. For interviews completed via zoom, we used CoreMedica HemaSpot-SE self-collection kits which participants mailed to a lab for processing.

### Overview of analysis

First, we compared the two groups on the background demographic and diabetes variables shown in Table 1. To the extent the two groups differed, those variables were used as covariates in all analyses. We conducted a 2 (group: romantic, non-romantic) by 2 (role: patient, dyad partner) repeated measures analysis of covariance to examine the effects of group, role, and the interaction between group and role on the two measures of communal coping and then the relationship and psychological outcomes. Because diabetes outcomes were only measured in patients, we examined group differences in those outcomes with a oneway analysis of covariance.

Finally, to determine whether communal coping was related to relationship and psychological outcomes for the two groups, we used actor-partner interdependence modeling (APIM) for distinguishable dyads (Kenny et al., 2006) to examine whether own communal coping (actor effect) and partner communal coping (partner effect) were related to outcomes. Actor effects reflect the link between communal coping and one’s own outcomes, which can occur for both patient and dyad partner. Partner effects reflect the link between partner communal coping and one’s own outcomes, which includes the effect of patient communal coping on dyad partners and the effect of dyad partner communal coping on patients. For these analyses, the first model included the previously mentioned covariates as well as effects for group (romantic = 0; nonromantic = 1) and role (patient = 0; dyad partner = 1). In the second model, we added four interaction terms: group by actor communal coping, group by partner communal coping, role by actor communal coping, and role by partner communal coping. In the absence of any significant interactions, we retained the first model. We ran these analyses twice, once for the appraisal aspect of communal coping and once for the collaboration aspect of communal coping, as previous research has empirically distinguished between the two (Zajdel & Helgeson, 2020), and they were only moderately related in this study. The results (standardized betas) are shown in Table 3.

Because diabetes outcomes only involved the patient, we used regression analysis to examine whether communal coping predicted outcomes by entering communal coping, group, and communal coping X group interactions into the model—first for appraisal and second for collaboration.

Given that the study was exploratory, we did not expect to be adequately powered to find small effects for group differences or even for actor and partner effects. For group comparisons, given the sample size (group *n*’s = 34 and 41; 75 dyads), alpha set at 0.05, and a two-tailed significance test, we did not have adequate power to detect small effects (*d* = 0.2), were somewhat underpowered to detect medium effects (*d* = 0.5; power = 0.56) but had adequate power to detect somewhat larger effects (*d* = 0.66; power = 0.80) and more than sufficient power to detect large effects (d = 0.80; power = 0.93). For the APIM analyses, we used Robert Ackerman’s shinyapps program (https://robert-a-ackerman.shinyapps.io/APIMPowerRdis/) and found that we did not have adequate power to detect small actor or partner effects (*d* = 0.2; power = 0.36), but had adequate power to detect somewhat larger effects (*d* = 0.35; power = 0.80) and more than sufficient power to detect medium effects (*d* = 0.5; power = 0.98).

## Results

### Identification of covariates

As shown in Table 1, the two groups differed on gender, such that 63% of the patients were men in the romantic couples, whereas 34% were men in the nonromantic couples; on insulin, such that 20% of patients in romantic couples were on insulin compared to 46% of patients in nonromantic dyads; on number of years with diabetes, such that patients in the nonromantic group had been diagnosed with diabetes longer than patients in the romantic group; on income, such that the patients in the nonromantic group had lower family income than patients in the romantic group. There were no group differences in age, education, and comorbidity, or work status. Thus, we statistically controlled for gender, insulin, number of years with diabetes, and family income in all analyses.

### Group comparisons on communal coping

As shown in Table 2, there were group differences in terms of both appraisal and collaboration. Patients and partners in romantic couples were more likely to say that diabetes was a shared problem and were more likely to collaborate with their dyad partner on diabetes than patients and partners in nonromantic couples. There were no effects of role or interactions between role and group.

### Group comparisons on psychosocial and diabetes outcomes

As shown in Table 2, the two groups differed on relationship intimacy but not relationship quality. Patients in the non-romantic group reported higher intimacy than patients in the romantic group. There was a role (patient vs. dyad partner) effect for relationship quality, such that patients reported higher relationship quality than dyad partners. There was a group difference in support, such that patients and dyad partners in romantic couples reported receiving more support from their partner than patients and dyad partners in non-romantic couples.

There were no group differences in psychological health or diabetes outcomes.

### APIM: benefits of communal coping by group

Regardless of the group differences in communal coping, we now turn to the question of whether communal coping has the same effects on outcomes for the two groups. To address this question, we examined the relation of appraisal first and collaboration second to the relationship and psychological health outcomes using actor-partner interdependence models.

#### Appraisal

As shown in Table 3, there was a main effect of role and a main effect of group for intimacy, such that patients reported higher intimacy than partners, and nonromantic couples reported higher intimacy than romantic couples, consistent with the previously reported findings. More importantly, there was an actor effect, such that own shared appraisal was related to one’s own relationship intimacy. None of the interactions were significant.

For the relationship quality index, there were main effects of actor and partner shared appraisal that each interacted with group. Both actor shared appraisal and partner shared appraisal were related to greater relationship quality for romantic couples (actor β = 0.33, SE = 0.14, *p* = 0.017; partner β = 0.45, SE = 0.14, *p* = 0.002) but unrelated to relationship quality for non-romantic couples (actor β = − 0.08, SE = 0.15, *p* = 0.589; partner β = − 0.14, SE = 0.16 *p* = 0.364). There was also an interaction of partner shared appraisal with role, such that partner shared appraisal was related to higher relationship quality for patients (β = 0.45, SE = 0.14, *p* = 0.002) but not dyad partners (β = − 0.02, SE = 0.15, *p* = 0.900).

Consistent with our earlier report, the romantic group reported greater support than the nonromantic group. Both actor and partner shared appraisal were related to greater support. There were no interactions of appraisal with group or role. There were no significant effects for either actor or partner shared appraisal for psychological health.

#### Collaboration

As shown in the bottom of Table 3, actor collaboration was related to higher intimacy, but there were no interactions with group or role. For relationship quality, there was a main effect of actor collaboration which was linked to higher relationship quality, as well as an interaction of partner collaboration with group. Partner collaboration was unrelated to relationship quality for the romantic group (β = 0.13, SE = 0.13, *p* = 0.312) but was related to lower relationship quality for the nonromantic group (β = − 0.21, SE = 0.14, *p* = 0.123); however, neither slope was statistically significant.

Actor collaboration was related to greater support and greater psychological health, but there were no interactions with group or role.

### Regression: predicting diabetes outcomes by group

There were no effects of appraisal or interactions of appraisal with group on any of the diabetes outcomes. Patient collaboration was related to lower diabetes distress (β = − 0.35, SE = 0.14, *p* = 0.011). There were no other effects of collaboration or interactions of collaboration with group on any of the diabetes outcomes.

## Discussion

The primary goal of this study was to examine whether communal coping extended from romantic relationships to non-romantic dyads among African Americans. We addressed this question in two ways. First, we compared levels of communal coping across the two groups. Then, we examined whether the relation of communal coping to outcomes was the same for the two groups.

Levels of communal coping—both shared appraisal and collaboration—were higher among romantic couples than nonromantic couples as reported by both patients and dyad partners. However, this does not mean that romantic couples were closer than nonromantic dyads, as nonromantic dyads reported higher levels of intimacy in their relationship compared to romantic couples, and there were no group differences in relationship quality. Yet, there was a greater exchange of support among romantic couples than non-romantic dyads. Communal coping may be more likely to occur and support provision may be more likely to transpire in the context of a romantic relationship than in the context of relationships with friends and family because there is the greater expectation to share problems and support one another. It also may be the case that romantic partners have greater opportunity for support provision than non-romantic dyads because they are living in the same household and presumably spending more time together. In addition, there is likely more variability in the nature of the relationship among the non-romantic dyads than the romantic couples. Unfortunately, we did not have a sufficient sample size to explore differences among the kinds of nonromantic dyads—for example, between friends and family.

These findings are important because there is a wealth of evidence that supportive interactions are related to better outcomes and unsupportive interactions are related to poorer outcomes in the context of diabetes. For example, one study of adult couples in which one person had diabetes showed that spouse encouragement was related to better patient adherence, but spouse warning (which may be perceived as controlling) was related to lower patient adherence (Stephens et al., 2010). Another study that examined persons with diabetes and their support persons found that harmful friend and family behaviors were related to more distress, whereas helpful behaviors were unrelated to distress (Roddy et al., 2023). Finally, a daily diary study showed that diet-related support was related to an increase in adherence, whereas diet-related persuasion and pressure were related to a decrease in adherence (Stephens et al., 2013). The latter study showed that communal coping plays a role in these relations: diet-related support was unrelated to diabetes distress on its own but was related to reductions in distress in the context of a high shared appraisal—that is, when patients perceived diabetes to be a shared problem.

In terms of the links between communal coping and relationship outcomes, there was substantial evidence that one’s own communal coping (whether patient or dyad partner) was related to better relationship outcomes—greater intimacy, higher relationship quality, and more support. This finding is consistent with a wealth of previous research (Helgeson et al., 2018) that shows communal coping draws couples closer together. And many of these effects generalized across romantic and non-romantic dyads, suggesting that communal coping may have benefits outside the context of a romantic relationship. This was one of the major questions that the present study asked. However, there was some nuance across these findings.

Appraising diabetes as a shared problem was related to higher relationship quality for romantic couples but not nonromantic dyads. One cannot conclude, however, that shared appraisal is not advantageous for nonromantic dyads because this interaction did not appear for relationship intimacy or for support exchanges. In addition, none of the benefits of collaboration were qualified by relationship type. More concerning is that the link of partner collaboration to relationship quality significantly differed between the two groups, with the direction being positive for romantic couples and negative for non-romantic couples; however, neither slope was significant. Nonetheless, these findings merit future investigation to determine if there are contexts in which communal coping creates a burden for some dyad partners.

In terms of whether patients or dyad partners reaped greater benefits from communal coping most of the findings here are consistent with previous research that both persons in the dyad benefit (e.g., Helgeson et al., 2022). The one finding that departs from this conclusion is that partner shared appraisal was linked to higher relationship quality for patients but not dyad partners. This finding is consistent with previous research. A meta-analysis that examined the links of both own versus partner shared appraisal on relationships and health concluded that partner shared appraisal had a stronger effect on patients than their own shared appraisal (Karan et al., 2019). We found in an earlier report that included this romantic sample that partner effects were more beneficial for patients than spouses (Helgeson et al., 2019b). These findings are not surprising, because whether the partner perceives diabetes to be a shared problem is likely to be a greater determinant of partner involvement in diabetes compared to whether patients perceive diabetes to be a shared problem. It is also the case that partners are more likely than patients to perceive diabetes to be a shared problem (Helgeson et al. 2017, 2019a, b). Although we did not find an effect of role (patient vs. partner) on shared appraisal, a post-hoc comparison of patient vs. partner shared appraisal among romantic couples showed that partners have a higher shared appraisal than patients, *t*(39) = 5.809, *p* < 0.001.

Whereas the links of communal coping to relationship outcomes were substantial, there was much less evidence for links of communal coping to health for either group. Collaboration was related to lower levels of psychological distress for both groups. However, there were no links of shared appraisal to diabetes outcomes, and only a single effect for collaboration. Collaborating with a partner was linked to lower levels of diabetes distress. However, these links to lower psychological and diabetes distress did not translate into better self-care or greater glycemic stability.

Before concluding, we note several study limitations. First and foremost, we considered this study to be exploratory. We were underpowered to detect small effects, which may explain why we did not find more communal coping by group interactions and may explain why we did not observe the effects on diabetes outcomes that have been found in other research (e.g., Zajdel & Helgeson, 2020). Because studies have not compared communal coping among romantic couples to relationships with family and friends, it is difficult to estimate what the likely effect size would be. The significant actor and partner effects we found for shared appraisal and collaboration ranged from small to large in terms of size, whereas the significant interactions were medium to large.

Second, we only examined this question among Black persons. Compared to White people, we thought Black people would be less likely to distinguish between romantic partners and non-romantic partners, as there is evidence that marriage plays a larger role in the lives of White compared to Black people (Cross et al., 2018) and that family and extended family play a larger role in the lives of Black compared to White people. Future research should examine this question among other racial/ethnic groups.

Third, although the recruitment methods for the sample in which the romantic couples were drawn and the new sample were the same (e.g., community advertising), the samples were not initially recruited at the same time which could have contributed to differences between the two groups. It is also important to note that the non-romantic group consisted of a higher proportion of women, were more likely to be on insulin, had diabetes for a longer period of time (likely related to being on insulin), and had lower income. We adjusted for these variables statistically, but these variables are substantive enough to consider the possibility that the non-romantic group differed in important demographic and disease-related ways that could account for the group differences we found. It is also important to note that about a third of the romantic couples were interviewed via zoom which could have affected these findings. Participants on zoom may have felt less connected to the interviewers which could have affected how they responded to interview questions. It is also the case that the romantic sample only included those who retained partners five years later, as 10 couples had separated or divorced. Thus, the romantic sample reflected those involved in more stable relationships.

In addition, it is a study limitation that shared appraisal was measured with only a single item. Although single-item measures of shared appraisal have been used in other studies that have revealed significant findings (e.g., Stephens et al., 2013), a multi-item measure would provide greater reliability and validity for the construct.

Finally, it is disappointing that the majority of benefits were observed for relationship rather than health outcomes. Given the importance of relationships for health (Robles et al., 2014; Uchino et al., 2018), it is somewhat surprising that health benefits were not observed. It may be that the effects on health are smaller than the effects on relationships. It also could be that the effects on relationships take time before benefits to health are observed.

In conclusion, although romantic couples engage in more communal coping than non-romantic dyads, communal coping was generally beneficial to relationships and psychological health for both groups of dyads. This finding is important because it suggests that the communal coping framework can be applied to people in one’s social network aside from a spouse. First, not all people have romantic partners, and among those who do, they do not always share living quarters. Second, for some couples there may other people in their network who are greater sources of support than their spouse. These findings have implications for the generalizability of communal coping theory and the potential for the development of communal coping interventions to affect both relationships and health.

**Table 1:** Demographics

Variable	Romantic n = 41	Non-romantic n = 34	*p* value
Gender	37% women; 63% men	68% women; 32% men	0.007
Race of partner	92.5% Black; 7.5% White	97% Black; 3% White	0.404
Relationship to partner	100% Romantic partner	38% Friend 35% Child 9% Sibling 6% Grandchild 6% Other family 3% Coworker 3% Neighbor	N/A
Education	M = 3.63; SD = 1.34	M = 3.71; SD = 1.29	0.815
Age	M = 54.67; SD = 10.83	M = 56.38; SD = 11.91	0.517
Comorbidity	M = 1.10; SD = 1.11	M = 1.41; SD = 2.02	0.396
Length of diabetes	M = 6.66; SD = 1.75	M = 10.17; SD = 5.07	0.001
Income+	M = 5.24; SD = 2.66	M = 3.02; SD = 1.57	0.001
Insulin	Yes 19.5%	Yes 44.1%	0.021
Currently working	Yes 51.2%	Yes 29.4%	0.056
Health insurance	Yes 97.6%	Yes 97.1%	0.893

n/a = not applicable; + Because 5 participants did not answer this question, we imputed income from the following variables: participant gender, education, work status, health insurance, comorbidity, use of insulin, and duration of diabetes; the income mean for the romantic sample corresponds to range of $50–59,999 and for the nonromantic sample of $30–39,999; N/A = not applicable

**Table 2:** Analyses of covariance: group comparisons on relationship, psychological health, and diabetes outcomes

Outcome variable	Romantic		Non-romantic				
	Patient M (SE)	Partner M (SE)	Patient M (SE)	Partner M (SE)	Group *p* value/ES	Person *p* value/ES	Group X Person *p* value/ES
Shared appraisal	2.49 (0.228)	4.15 (0.208)	2.04 (0.251)	3.03 (0.219)	0.003/0.125	0.881/0.000	0.169/0.028
Collaboration	0.50 (0.122)	0.38 (0.136)	− 0.58 (0.136)	− 0.47 (0.152)	< 0.001/0.325	0.156/0.029	0.380/0.011
Intimacy	5.57 (0.168)	5.03 (0.193)	6.26 (0.188)	6.10 (0.215)	< 0.001/0.165	0.052/0.053	0.448/0.008
Relationship quality	6.17 (0.158)	5.98 (0.183)	5.67 (0.177)	5.75 (0.204)	0.117/0.035	0.027/0.069	0.411/0.010
Support	1.76 (0.125)	1.72 (0.122)	1.05 (0.140)	1.19 (0.136)	< 0.001/0.174	0.852/0.001	0.439/0.009
Psychological health	− 0.07(0.142)	− 0.05 (0.140)	0.08 (0.159)	0.06 (0.157)	0.495/0.007	0.644/0.003	0.861/0.000
Diabetes distress	2.00 (0.142)		2.17 (0.159)		0.463/0.008		
Self-care	− 0.018 (0.098)		− 0.004 (0.109)		0.934/0.000		
Medication adherence	4.02 (0.172)		4.27 (0.150)		0.309/0.019		
HbA1c	7.41 (0.296)		8.03 (0.316)		0.198/0.025		

These analyses control for income, insulin delivery method, gender, and diabetes duration. ES: Effect size

**Table 3:** Actor partner interdependence model results

	Intimacy	Relationship Quality	Support	Psychological Health
	β (SE)	β (SE)	β (SE)	β (SE)
**Shared appraisal**				
Income	0.23* (0.09)	0.18 (0.11)	− 0.23* (0.09)	0.31** (0.11)
insulin delivery	0.59** (0.18)	0.52* (0.21)	0.53** (0.18)	0.20 (0.22)
Gender	− 0.16 (0.14)	− 0.17 (0.14)	− 0.28* (0.14)	− 0.19 (0.15)
Diabetes duration	0.05 (0.09)	0.05 (0.10)	− 0.04 (0.09)	− 0.04 (0.11)
Actor SA	0.22** (0.08)	0.33* (0.14)	0.33*** (0.08)	0.18+ (0.09)
Partner SA	0.03 (0.08)	0.45** (0.14)	0.23** (0.08)	0.07 (0.09)
Role	− 0.49** (0.16)	− 0.26 (0.16)	− 0.01 (0.16)	− 0.04 (0.17)
Group	0.85*** (0.19)	− 0.14 (0.22)	− 0.48* (0.20)	0.23 (0.23)
Actor SA X Role		0.10 (0.18)		
Partner SA X Role		− 0.47** (0.18)		
Actor SA X Group		− 0.41* (0.17)		
Partner SA X Group		− 0.60*** (0.17)		
**Collaboration**				
Income	0.28** (0.09)	0.21 (0.11)	− 0.08 (0.07)	0.37* (0.11)
Insulin delivery	0.51** (0.17)	0.49* (0.21)	0.25 (0.14)	0.01 (0.21)
Gender	− 0.08 (0.13)	− 0.15 (0.14)	− 0.12 (0.11)	− 0.18 (0.05)
Diabetes duration	0.06 (0.09)	0.04 (0.10)	− 0.02 (0.07)	− 0.02 (0.11)
Actor collab	0.36*** (0.08)	0.50*** (0.14)	0.69*** (0.06)	0.25** (0.09)
Partner collab	− 0.08 (0.08)	0.13 (0.13)	0.12 (0.06)	0.10 (0.09)
Role	− 0.32** (0.12)	− 0.05 (0.13)	0.08 (0.10)	0.03 (0.13)
Group	1.04 (0.21)	− 0.00 (0.25)	0.10 (0.16)	0.53* (0.25)
Actor collab X Role		− 0.12 (0.18)		
Partner collab X Role		0.02 (0.19)		
Actor collab X Group		− 0.28 (0.17)		
Partner collab X Group		− 0.34* (0.16)		

+ = 0.055, * *p* < .05; ** *p* < .01; *** *p* < .001; SA: Shared appraisal; Collab: Collaboration; Gender is coded 1 = male; 2 = female; Role is coded 0 = patient and 1 = dyad partner; Group is coded 0 = romantic and 1 = nonromantic; continuous variables were grand-mean centered to obtain standardized regression coefficients

## Data Availability

All materials are available at https://osf.io/zm3ew/.
